# Weight stigma and mental health symptoms: mediation by perceived stress

**DOI:** 10.3389/fpsyt.2025.1587105

**Published:** 2025-07-02

**Authors:** David G. Figueroa, William D. Murley, Jordan E. Parker, Jeffrey M. Hunger, A. Janet Tomiyama

**Affiliations:** ^1^ Department of Psychology, University of California, Los Angeles, Los Angeles, CA, United States; ^2^ Department of Psychology, Miami University, Oxford, OH, United States

**Keywords:** weight stigma, perceived stress, mental health psychological symptoms, depressive symptoms, anxiety symptoms

## Abstract

Prior research has established that weight stigma, or social devaluation based on an individual’s body size or weight, is directly related to greater depressive and anxiety symptoms. In this investigation, we apply the Cyclic Obesity/Weight-Based Stigma model to investigate if the association between weight stigma and poor mental health is mediated by greater perceived stress. We analyzed data from a census-matched sample (N=1,993) of the U.S. on age, race/ethnicity, gender, income, and census-region. Issues with missing data and mediation models were addressed using a Bayesian multiple imputation approach. Analyses controlled for Body Mass Index and sociodemographic variables as covariates. Weight stigma was directly associated with greater depressive and anxiety symptoms. Moreover, the relationship between weight stigma and greater depressive and anxiety symptoms was mediated by greater perceived stress. Perceived stress explained 37% of the relationship between weight stigma and mental health outcomes, even after accounting for Body Mass Index. These results provide evidence for weight stigma as an important psychosocial stressor that contributes to poor mental health outcomes.

## 1 Introduction

Weight stigma is defined as the social devaluation of individuals based on their body size or weight, often displayed as prejudice, stereotyping, and discrimination (1). Some work has reported that more than 50% of larger-bodied U.S. adults experience weight stigma (2), but others have observed even higher prevalence estimates much closer to 100% (3, 4). Experiencing weight stigma can incur negative consequences, including poor mental health. Indeed, multiple systematic reviews and meta-analyses document the negative impact weight stigma can have on psychological health including depressive and anxiety symptoms. Crucially, these associations remain even while accounting for Body Mass Index (BMI), suggesting that weight stigma explains a significant amount of variance in mental health symptoms and is not confounded by higher BMI individuals merely experiencing more weight stigma and poorer outcomes (5–7). However, research examining factors that mediate or explain these relationships is relatively limited.

**Figure 1:**
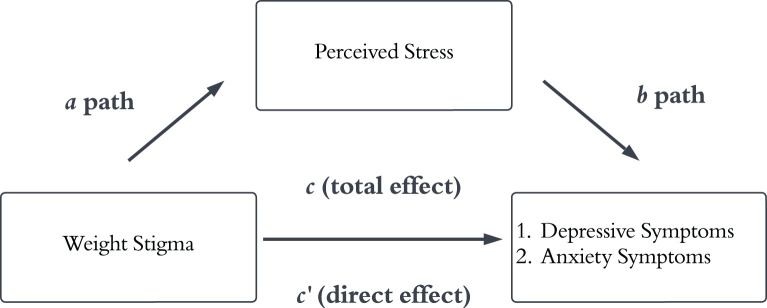
Conceptual model depicting the direct and indirect pathways for two respective mediation analyses. The *a* path depicts the relationship between weight stigma and greater perceived stress. The *b* path depicts the relationship between greater perceived stress and depressive and anxiety symptoms, respectively. The *c’* path depicts the direct relationship between weight stigma and depressive and anxiety symptoms.

Thus far, constructs like eating disturbances (8), internalized weight stigma (9), and social identification with higher-weight groups (10) have been shown to significantly mediate the relationship between weight stigma and mental health symptomatology. In this investigation, we hypothesize that a key overlooked mediator is perceived stress (**Figure 1**).

Weight stigma has been characterized as a psychosocial stressor under the Cyclic OBesity/WEight-Based Stigma (COBWEBS) model (1). The COBWEBs model posits that stress arising from weight stigma trigger changes in eating behaviors and increases in cortisol that contribute to weight gain. The model is characterized as a positive feedback loop, as subsequent weight gain may place an individual at greater risk for future weight stigma (1). Similar theoretical work suggests that both direct experiences of weight stigma and the anticipation of future stigma can trigger social identity threat that is linked to greater psychological stress (11). The relationship between weight stigma and negative psychological outcomes, including greater perceived stress and greater distress, is well established. For instance, one investigation reported that higher weight women, compared to average weight women, reported greater stress-related emotions when their weight was visible to others (12). A systematic review of 23 studies assessing the psychological correlates of weight stigma among adults with obesity or overweight reported that weight stigma was significantly correlated with greater perceived stress (13). A recent meta-analysis of 30 studies reported similar findings, with weight stigma being moderately associated with greater psychological distress (5). These findings substantiate the hypothesized *a* path wherein experiences with weight stigma are positively associated with perceived stress.

Prior work has also provided evidence for the hypothesized *b* path where greater perceived stress exacerbates mental health symptomology. For instance, a meta-analysis of data from the World Health Survey, which included 232,243 individuals from middle- and low-income countries, found that each one-unit increase on the perceived stress scale was associated with 1.4 greater odds of depression (14). Similar patterns have been observed for anxiety symptoms. One investigation that included mentally healthy individuals and patients with major depressive disorder reported a positive linear relationship between stress levels and anxiety, regardless of the severity of the stress levels reported (15).

To our knowledge, no study has yet examined the mediating role of perceived stress in the relationship between weight stigma and mental health symptomatology within a single investigation. Therefore, the current study leveraged data from a census-matched U.S. sample to test two hypotheses (see **Figure 1**). First, we hypothesized that weight stigma would be directly and positively associated with greater depressive and anxiety symptoms, respectively. Second, we hypothesized that greater perceived stress would mediate the relationship between weight stigma and depressive and anxiety symptoms. This study was preregistered on the Open Science Framework: https://osf.io/xve7j/?view_only=d58c8d92ebca400e859276634c127cf8.

## 2 Materials and methods

### 2.1 Participants and procedure

The sample (*N*=2,022) was census-matched using quotas based on U.S. Census benchmarks for age, gender, race/ethnicity, income, and census region. Participants provided informed consent and completed self-report measures administered on Qualtrics between December 2019 and January 2020. All study materials and procedures were approved by the university’s Institutional Review Board. Responses were excluded from the analytic sample if participants (a) failed attention checks; (b) reported implausible height (≤44 inches or ≥90 inches) or weight (≤55 pounds or ≥1000 pounds); or (c) displayed BMIs less than 12 or greater than 70. The final analytic sample for this study consisted of 1,993 respondents (*M*_age_=47.22, *SD*=17.29). Sample demographics are displayed in **Table 1**.

**Table 1:** Sample characteristics.

Characteristic	*n*	%
Gender		
Woman	1,023	51.33%
Man	965	48.42%
Non-binary/Other term	5	0.25%
Race/Ethnicity		
Asian/Asian-American	103	5.17%
Black/African-American	263	13.20%
Hispanic/Latino(a)	309	15.50%
Indigenous, Alaskan Native, or Aleut	25	1.25%
Native Hawaiian/Pacific Islander	2	0.10%
White	1255	62.97%
Biracial/Multiracial	28	1.40%
Other	8	0.40%
Education level		
Less than High School	37	1.86%
High School Diploma or equivalent (e.g., GED)	353	17.71%
Some college, but not degree	442	22.18%
Associate Degree	238	11.94%
Bachelor’s Degree	430	21.58%
Master’s Degree	201	10.09%
Doctorate or Professional Degree (e.g., JD, MD)	65	3.26%
Income		
< $25,000	362	18.16%
$25,000 - $49,999	448	22.48%
$50,000 - $74,999	384	19.27%
$75,000 - $99,999	288	14.45%
$100,000 - $149,999	289	14.50%
$150 - $199,999	112	5.62%
> $200,000	110	5.52%
Region		
Northeast	357	17.91%
South	732	36.73%
Midwest	453	22.73%
West	451	22.63%

Frequencies and percentages were calculated using non-imputed data. Missing data for education level (*n*=227).

Income is presented as a categorical variable in the table but is included as a continuous variable in mediation models.

### 2.2 Measures

#### 2.2.1 Weight stigma

Experienced weight stigma was assessed using a single item adapted from Williams et al. (1997; “How often are you treated with less respect, harassed, or discriminated against because of your weight?”) (16). Anticipated weight stigma was assessed using a single item from Hunger and Major (2015; “How often are you concerned about or worried that you will be negatively stereotyped or mistreated because of your weight?”) (17). Participants responded on a 4-point scale (Not at all - Often), with higher scores indicating higher levels of weight stigma. Scores from the experienced and anticipated weight stigma items were averaged to create a single composite weight stigma score. The weight stigma composite had good internal consistency (α=.85) and the two items were highly correlated (*r*=.75). Previous research with this sample demonstrated that the composite weight stigma measure was strongly correlated with other validated weight stigma questionnaires (18).

#### 2.2.2 Perceived stress

Perceived stress was assessed using a 4-item Perceived Stress Scale (e.g., “How often have you felt nervous and stressed?”) (19). Participants reported perceived stress over the past month on a 5-point scale (Never-Very Often). Responses were averaged with larger scores indicating higher levels of perceived stress (*α*=.71).

#### 2.2.4 Depressive symptoms

Depressive symptoms in the last seven days was assessed using the 4-item PROMIS depressive symptoms short form (e.g., “Little interest or pleasure in doing things.”) (20). Items were presented on a 4-point scale (Not at all - Nearly Every Day). Responses were averaged with greater scores representing higher levels of depressive symptoms (*α*=.94).

#### 2.2.3 Anxiety symptoms

Anxiety symptoms in the last seven days were assessed with the Patient-Reported Outcomes Measurement Information System (PROMIS) anxiety 4-item measure (e.g., “I found it hard to focus on anything other than my anxiety.”) (21). Items were presented with a 5-point scale (Never - Always). Items were averaged such that higher scores reflected higher levels of anxiety symptoms (*α*=.93).

#### 2.3 Analytic approach

A G*Power analysis (22) indicated that a sample size of *N* = 1,043 would be sufficient to detect a small effect size (*f*² =.02, α=.05, =.80) with 19 predictors. Missing data (range 0–11.79%) was addressed using a Bayesian model-based imputation procedure (23). This robust strategy imputes missing data by relying on auxiliary variables that are correlated with missingness, model residuals, or both. Statistical analyses were conducted using the *rblimp* package within R 4.1.0. Two separate mediation models tested our hypotheses by assessing the significance of the conditional direct and indirect effects of weight stigma on depressive and anxiety symptoms, indirectly via perceived stress. Results were deemed significant if the 95% credible interval did not contain a null value of zero. Factors including age, BMI, census region, gender, income, education, and race/ethnicity were included as covariates. Census region, gender, education, and race/ethnicity were dummy-coded. Covariates were selected based on previous research with the same dataset (18, 24).

Exploratory multiple linear regression was used to examine the associations of sociodemographic factors (age, BMI, census region, gender, income, education, race/ethnicity) with weight stigma as an outcome. Results are presented in **Supplementary Table 1**.

## 3 Results

### 3.1 Partial correlations

Partial correlations were calculated between the focal variables (weight stigma, perceived stress, anxiety symptoms, and depressive symptoms) with age, BMI, and income included as covariates (**Table 2**). Weight stigma was positively related to perceived stress, anxiety symptoms, and depressive symptoms. Perceived stress was positively associated with anxiety and depressive symptoms. Anxiety symptomatology was positively correlated with depressive symptoms.

**Table 2:** Partial correlations table for focal variables.

Measure	1	2	3	4
1. Weight Stigma	–			
2. Perceived Stress	.25^***^	–		
3. Depressive Symptoms	.38^***^	.66^***^	–	
4. Anxiety Symptoms	.37^***^	.62^***^	.77^***^	–
Descriptives *M*(*SD*)	1.77 (0.86)	2.66 (0.83)	1.17 (1.12)	2.13 (1.05)

Partial correlations were computed using non-imputed data. Age, BMI, and income were included as covariates.

Missing data for weight stigma (*n*=149), perceived stress (*n*=148), depressive symptoms (*n*=161), and anxiety symptoms (*n*=169).

Bold indicates statistical significance, ^***^ *p*<.001.

### 3.2 Anxiety symptoms

Weight stigma was significantly directly associated with anxiety symptoms (**Table 3**). Furthermore, a significant indirect effect was reported, indicating that perceived stress significantly mediated the relationship between weight stigma and anxiety, even after controlling for BMI and other covariates. Perceived stress explained roughly 37% of the relationship between weight stigma and anxiety symptoms.

**Table 3:** Results from mediation models testing perceived stress as a mediator between weight stigma and mental health symptoms.

Outcome	*a*(*SD*)	95% CI	*b*(*SD*)	95% CI	Indirect Effect (*SD*)	95% CI	*c’*(*SD*)	95% CI	*c*(*SD*)	95% CI	% Mediated
Depression Symptoms	**0.24(0.02)**	**0.20,0.29**	**0.79(0.02)**	**0.75,0.84**	**0.19(0.02)**	**0.16,0.23**	**0.31(0.02)**	**0.26,0.35**	**0.50(0.03)**	**0.44,0.56**	**38%**
Anxiety Symptoms	**0.24(0.02)**	**0.20,0.29**	**0.70(0.02)**	**0.65,0.74**	**0.17(0.02)**	**0.14,0.20**	**0.29(0.02)**	**0.25,0.34**	**0.46(0.03)**	**0.41,0.52**	**37%**

*a=a* path, *b*=*b* path*, c’*= direct effect, *c*=total effect, 95% CI=95% credible interval, *SD*=standard deviation.

Bold indicates significant effect; 95% CI does not contain zero.

### 3.3 Depressive symptoms

Weight stigma was significantly directly associated with depressive symptoms (**Table 3**). Furthermore, a significant indirect effect was observed, suggesting that perceived stress significantly mediated the relationship between weight stigma and depressive symptoms while holding BMI and other covariates constant. Perceived stress explained roughly 38% of the relationship between weight stigma and depressive symptoms.

### 3.4 Exploratory analyses

Multiple linear regression models indicated that age was negatively associated with weight stigma. Models suggested that BMI was positively associated with weight stigma. Additionally, Hispanic/Latino and Asian/Asian American participants reported significantly less weight stigma than Black/African American participants (reference group).

## 4 Discussion

This study aimed to investigate relationships between weight stigma and depressive and anxiety symptoms using a census-matched U.S. sample. The chief contribution of the current study is that it tested whether perceived stress functioned as a mediator of these relationships. As hypothesized, weight stigma was directly positively associated with both mental health outcomes. This finding is consistent with existing literature, providing additional evidence of the negative mental health impacts weight stigma may elicit (5–7). Furthermore, the significant indirect effects of perceived stress in both models aligned with and complemented the COBWEBS model, suggesting that stress is a crucial mechanism through which weight stigma influences psychological health. We observed that perceived stress explained 37% of the relationship between weight stigma and mental health symptoms, highlighting the potential utility of addressing stress when considering interventions aimed at mitigating the consequences of weight stigma.

We note several strengths of the current study. Our hypotheses were tested using a large, census-matched sample of the U.S. and thus results from this study have high generalizability to the U.S. population. Potential bias introduced with missing data issues was mitigated using a rigorous Bayesian statistical approach. Associations between weight stigma, mental health symptoms, and perceived stress remained significant even after accounting for BMI. These results suggest that the observed relationship was not explained by body size alone, highlighting that weight stigma is a unique psychosocial stressor that may contribute to poor mental health symptoms.

### 4.1 Limitations and future directions

The cross-sectional nature of the data did not allow for causal inferences. While significant relationships between the focal variables controlled for likely confounds, no causal conclusions can be made about weight stigma causing greater perceived stress, anxiety, or depressive symptoms. When testing mediation cross-sectionally, researchers must provide compelling evidence for the temporal ordering tested (25). The mediation model we propose is well-justified given the existing experimental work has established that weight stigma causes increases in stress (e.g (26, 27)) and stress causes increases in depressive and anxiety symptoms (e.g (28, 29)). While the results do not establish causal relationships among the focal variables, they offer preliminary evidence suggesting that perceived stress may function as an exploratory mechanism. Experimental work will be useful for establishing that instances of weight stigma cause changes in mental health in participants. Longitudinal designs will also be helpful to substantiate the mediation models and confirm the temporal order of these variables.

Another limitation is that brief measures were used to assess mental health symptomology due to time constraints and participant burden. Future work may consider replicating these findings with comprehensive mental health assessments, including diagnostic interviews. Moreover, self-reported measures, particularly those related to sensitive topics such as weight stigma, anxiety, and depressive symptoms, may be susceptible to social desirability bias (30). It is possible that some participants may have underreported these behaviors to conform to perceived social norms. Future research may consider incorporating objective biomarkers of stress, such as cortisol, to complement self-reported data and provide a more comprehensive understanding of the relationship between weight stigma and mental health. Stressors like weight stigma can influence the hypothalamic-pituitary-adrenal axis to release cortisol—originally serving to help the body cope with acute stress, but with chronic or repeated exposure to stressors, cortisol levels and response functioning may become dysregulated. Studies suggest cortisol dysregulation can increase inflammation (31), interfere with sleep (32), and impact regions of the brain such as the prefrontal cortex and hippocampus (33). Although confirmation is needed in future studies, these factors, among many others, could collectively elevate the risk for anxiety and depressive symptoms via psychological stress. Lastly, there are additional factors that are likely to influence levels of perceived stress and mental health symptoms in addition to weight stigma that were not accounted for in this study. Comorbid health conditions, previous experiences with other forms of discrimination, and internalized weight stigma may all be associated with greater stress and contribute to worse mental health outcomes. Similarly, although race, income, and education were statistically controlled for, these structural and socioeconomic factors are important independent determinants of perceived stress and mental health.

### 4.2 Conclusion

The current study contributes to the growing body of evidence linking weight stigma to adverse mental health outcomes, namely anxiety and depressive symptoms. Our findings support the COBWEBS model by demonstrating that perceived stress accounts for up to 38% of the relationship between weight stigma and mental health symptomatology. The indirect effect of perceived stress on depressive and anxiety symptoms was a relatively small effect (υ=.02) per Cohen’s benchmarks for proportion of variance explained (34). Although the effect size is small, such effects can accumulate over time or across larger populations, potentially leading to significant changes in mental health symptoms. If replicated in future experimental and longitudinal studies, these findings indicate the need for stress-reduction strategies in interventions aimed at individuals experiencing weight stigma. Existing work on mindfulness-based interventions show promise for mitigating weight stigma related stressors and improving mental health and affect (35, 36). Additionally, the implications of this study extend beyond clinical practice, suggesting the need for public health policies that address the broader societal contributors to weight stigma, reducing its potential harmful psychological impact at a population level.

## Data Availability

The original contributions presented in the study are included in the article/**Supplementary Material**. Further inquiries can be directed to the corresponding author.
