# Supplementary material for: Weight stigma and mental health symptoms: mediation by perceived stress
Source: Front Psychiatry. 2025 Jul 2;16:1587105. doi: 10.3389/fpsyt.2025.1587105 (PMC12265077; doi:10.3389/fpsyt.2025.1587105)
Supplement: Supplementary file 1 [file Table1.docx]

Supplementary Table 1.

Associations between sociodemographics and weight stigma.

| Variable | Estimate | *SD* | 95% CI |
| --- | --- | --- | --- |
| **Age** | **-.02** | **.001** | **-0.02, -0.01** |
| **Body Mass Index** | **0.04** | **0.003** | **0.04, 0.05** |
| Income | -0.01 | 0.01 | -0.03, 0.02 |
| Race/Ethnicity |  |  |  |
| **Hispanic/Latino/a/x** | **-0.35** | **0.18** | **-0.69, -0.01** |
| **Asian/Asian American** | **-0.21** | **0.09** | **-0.39, -0.02** |
| Indigenous, Alaskan Native, or Aleut | -0.17 | 0.09 | -0.36, 0.009 |
| Native Hawaiian/Pacific Islander | -0.15 | 0.18 | -0.51, 0.21 |
| White | -0.86 | 0.56 | -1.95, 0.23 |
| Biracial/Multiracial | -0.10 | 0.31 | -0.70, 0.49 |
| Other | -0.12 | 0.09 | -0.29, 0.05 |
| Gender |  |  |  |
| Woman | 0.07 | 0.04 | -0.007, 0.15 |
| Non-binary/Other | -0.13 | 0.35 | -0.80, 0.57 |
| Education |  |  |  |
| High school diploma or equivalent (GED) | 0.09 | 0.14 | -0.18, 0.36 |
| Some college, but no degree | 0.09 | 0.14 | -0.18, 0.36 |
| Associate degree | 0.14 | 0.14 | -0.15, 0.41 |
| Bachelor’s degree | 0.11 | 0.14 | -0.17, 0.38 |
| Master’s degree | 0.16 | 0.15 | -0.13, 0.46 |
| Doctorate or Professional Degree (JD, MD) | 0.18 | 0.17 | -0.16, 0.51 |
| Region |  |  |  |
| South | 0.04 | 0.05 | -0.07, 0.14 |
| Midwest | -0.01 | 0.06 | -0.13, 0.11 |
| West | -0.04 | 0.06 | -0.15, 0.08 |

*Note*. *SD*=Standard deviation, 95% CI = 95% Credible interval

Estimates are based on 10000 iterations using 20 chains.

Missing data for education (*n*=227).

Race and ethnicity, gender, education, and region were dummy coded. Black/African American was the reference group for race/ethnicity. Man was the reference group for gender. Less than high school was the reference group for education. Northeast was the reference group for region.

Estimate is significant if the 95% CI does not contain zero.
